# PlexinA1 is crucial for the midline crossing of callosal axons during corpus callosum development in BALB/cAJ mice

**DOI:** 10.1371/journal.pone.0221440

**Published:** 2019-08-20

**Authors:** Md. Mosharaf Hossain, Takamasa Tsuzuki, Kazuki Sakakibara, Fumitaka Imaizumi, Akihiro Ikegaya, Mami Inagaki, Ikuko Takahashi, Takuji Ito, Hyota Takamatsu, Atsushi Kumanogoh, Takayuki Negishi, Kazunori Yukawa

**Affiliations:** 1 Department of Physiology, Faculty of Pharmacy, Meijo University, Nagoya, Japan; 2 Radioisotope Center, Faculty of Pharmacy, Meijo University, Nagoya, Japan; 3 Department of Immunopathology, Immunology Frontier Research Center, Osaka University, Suita, Japan; University of Queensland, AUSTRALIA

## Abstract

The corpus callosum (CC) is the biggest commissure that links cerebral hemispheres. Guidepost structures develop in the cortical midline during CC development and express axon guidance molecules that instruct neurons regarding the proper direction of axonal elongation toward and across the cortical midline. Neuropilin-1 (Npn1), a high affinity receptor for class 3 semaphorins (Sema3s) localized on cingulate pioneering axons, plays a crucial role in axon guidance to the midline through interactions with Sema3s. However, it remains unclear which type of Plexin is a component of Sema3 holoreceptors with Npn1 during the guidance of cingulate pioneering axons. To address the role of PlexinA1 in CC development, we examined with immunohistochemistry the localization of PlexinA1, Npn1, and Sema3s using embryonic brains from wild-type (WT) and PlexinA1-deficient (PlexinA1 knock-out (KO)) mice with a BALB/cAJ background. The immunohistochemistry confirmed the expression of PlexinA1 in callosal axons derived from the cingulate and neocortex of the WT mice on embryonic day 17.5 (E17.5) but not in the PlexinA1 KO mice. To examine the role of PlexinA1 in the navigation of callosal axons, the extension of callosal axons toward and across the midline was traced in brains of WT and PlexinA1 KO mice at E17.5. As a result, callosal axons in the PlexinA1 KO brains had a significantly lower incidence of midline crossing at E17.5 compared with the WT brains. To further examine the role of PlexinA1 in CC development, the CC phenotype was examined in PlexinA1 KO mice at postnatal day 0.5 (P0.5). Most of the PlexinA1 KO mice at P0.5 showed agenesis of the CC. These results indicate the crucial involvement of PlexinA1 in the midline crossing of callosal axons during CC development in BALB/cAJ mice.

## Introduction

Neuronal interhemispheric connections and commissures composed of midline traversing axons are needed for the mammalian brain to synchronize activities between contralateral regions [1]. The corpus callosum (CC) is the largest commissural tract and is composed of large bundles of axons interconnecting the two cerebral hemispheres [2]. The human CC contains 200–350 million axons [3] and represents a crucial pathway for the functional integration of cognitive and sensory information by mutually transferring information between the two cerebral hemispheres [4]. Some individuals with agenesis of CC (AgCC) are asymptomatic, but many cases are associated with hydrocephalus, mental retardation, microcephaly, seizures, and other abnormalities [5]. Individuals with AgCC experience various difficulties in physical, sensory, developmental, and motor aspects that cannot be easily explained by the existence of conditions such as low IQ scores, neurological disorder, and impairment of the age-level communication [6]. AgCC is also associated with major psychiatric disorders including autism, attention deficit hyperactivity disorder, and schizophrenia [7, 8, 9]. Thus, it is necessary to elucidate the mechanism of CC development to better understand the pathogenesis of disorders related to AgCC and to develop novel therapeutics according to its pathology [10, 11, 12, 13]. Many similarities in the developmental processes of CC have been observed in humans and mice, and structures of the midline glia as well as the expression profiles of genes involved in CC formation in humans have remarkable similarities with those in mice [14]. Mouse models are thus useful for investigating the mechanisms of CC development [15, 16]. In the midline region of the brain in mice, the CC begins to develop on approximately embryonic day 15 (E15) and continues developing until postnatal day 14 (P14) [15, 17, 18]. Many molecules are known to participate in the guidance of axons from the neocortex across to the CC [1, 19]. Axon guidance molecules involved in this process are Netrins [20, 21, 22], Slits [21, 23, 24], Semaphorins [19, 20, 21, 25, 26], Ephrins [21, 27, 28], and Draxin [29], while the implicated axon guidance receptors are DCC [30], Ryk [31], Robo [32], and Neuropilin [19]. Furthermore, guidepost structures including a glial wedge, indusium griseum glia, midline zipper glia, and subcallosal sling positioned laterally, dorsally, and ventrally to the CC may provide a guidance substratum for navigating the extension of callosal axons [1, 33, 34, 35].

The direction of neuronal axon elongation is determined by the actions of axon guidance molecules, which exert attractive or repulsive activities on the extension of axons through their receptors [20, 21]. Semaphorins (Semas) are involved in determining the direction of axonal extension. Among class3 semaphorins (Sema3s), Sema3C exhibits attractive activity toward growing axons and Sema3A displays repulsive activity toward extending axons [19, 36, 37]. Plexins and Neuropilin (Npn) have been identified as receptors for Semas [38, 39, 40, 41, 42]. Plexins function as principal bodies for transducing Semas’ signals intracellularly and consists of four classes, namely PlexinA, B, C, and D [38, 39, 40, 41]. Npn, a transmembrane receptor with high affinity to Sema3s, consists of Npn1 and Npn2 and interacts with plexins to transduce the signal of Sema3s [38, 39, 40, 41, 42, 43]. PlexinA1 is considered to transduce signals of Sema3s by forming a receptor complex with Npn1 on the growth cone of the axon terminals of developing neurons [38, 39, 44, 45].

During guidance of the formation of axonal pathways in the nervous system, pioneer neurons first project their axons toward the direction of their target neurons along the neuronal pathway, and axon tracts are formed thereafter [19]. These axons are called pioneer or pioneering axons [19]. During CC development in mice, pioneer axons derived from the cingulate cortex extend toward the rostral midline on embryonic day 15.5 (E15.5), opening a path for later-arriving neocortical axons [46, 47, 48]. Cingulate pioneering axons, which cross the midline prior to axonal formation in the CC, are reported to express Npn1 [19]. These cingulate pioneering axons are misguided into the septum in the knock-in mice expressing mutant Npn1 to which Sema3s are unable to bind, proving that Npn1 is necessary for the guidance of these axons across the cortical midline [19]. Sema3C attracts cingulate axons toward the midline by binding Npn1 expressed on the axons [19, 25]. Calretinin (CR)-positive glutamatergic neurons (CR+ neurons) expressing the calcium-binding CR protein emerge in the cortical midline in the early stages of CC development and produce Sema3C [25]. Sema3C secreted by CR neurons stimulates the elongation of cingulate axons with the expression of the receptor neuropilin1 (Npn1) toward the midline [25]. Both CR neurons and Sema3C are colocalized to the same areas in the midline, and CR neurons with Sema3C greatly contribute to CC development by guiding callosal axons to the midline [25]. After Semas bind to Npn1, the signals are supposed to be transmitted intracellularly through class A plexins (PlexinA1, A3, and A4), PlexinD1, or L1CAM, a component of the Sema3 holoreceptor [19, 25, 49, 50]. However, it remains unclear which molecules transduce Semas signals intracellularly to guide pioneer and callosal axons to the midline [51].

To gain insight into the role of PlexinA1 in CC development, the present study was conducted to examine the expression of PlexinA1 and Npn1 as well as ligands such as Sema3A and Sema3C involved in the developmental stage of CC in WT and PlexinA1 knock-out (KO) mice. The status of the guidance of Npn1-positive (Npn1+) callosal axons to and across the midline on E17.5 was then compared between brains of WT and PlexinA1 KO mice. Furthermore, we compared the status of CC formation between WT and PlexinA1 KO brains at P0.5 by examining the midline crossing of L1CAM+ callosal axons. Based on the results, we discuss the role of PlexinA1 in the CC development of BALB/cAJ mice.

## Materials and methods

### Animals

Animals used in this study research were PlexinA1 WT and PlexinA1 KO mice with a BALB/cAJ genetic background. PlexinA1 KO mice were produced by gene targeting with E14.1 embryonic stem (ES) cells [52]. The gene targeting vector was constructed to replace the genomic region containing the initiation codon and the Sema domain-coding sequence with a neomycin-resistance gene, and then were transfected into E14.1 ES cells by electroporation. G418-and ganciclovir-resistant clones were selected by polymerase chain reaction (PCR) and confirmed by Southern blotting. Mutant ES cells were introduced into mouse blastocysts and transferred into pseudo-pregnant mice to generate chimeras. F1 heterozygous KO mice were generated by breeding the chimeras with BALB/cAJ mice (CLEA Japan, Inc. Japan) and were backcrossed 10 generations to BALB/cAJ mice. Pairs of the resultant heterozygous mice were bred to obtain homozygous KO mice and their WT littermates as controls [53]. The mice were reared in the animal center in the Faculty of Pharmacy at Meijo University. The care and use of mice as well as other experimental protocols were performed in accordance with the guidelines promulgated by the Physiological Society of Japan and the guidelines on animal experimentation of the Meijo University. The Animal Ethics Review Committee of Meijo University approved the experimental protocols (authorization number: 2019PE4).

### Generation of Embryos and neonatal mice

PlexinA1 heterozygous mice were mated to obtain WT and PlexinA1 KO embryos and neonatal mice at postnatal day 0.5 (P0.5). Embryonic day 0.5 (E0.5) was specified as the day when the experimenter confirmed the presence of a vaginal plug. Embryos were obtained from the pregnant mice and neonatal mice were collected at P0.5 just after birth through the close monitoring of delivery.

### Genotyping

The genotypes of both adult mice and their offspring were identified by PCR using mouse tail DNA and a PlexinA1 gene specific primer set as previously reported [52, 53].

### Preparation of Embryos and neonatal mice

Embryos were collected from the uterus of the pregnant mice under anesthesia. The heart of the embryo was visualized after removal of the skin and thoracic muscles. Then, transcardial perfusion of phosphate-buffered solution (PBS) at pH 7.4 and successive perfusion of 4% paraformaldehyde solution (PFA) in PBS at pH 7.4 were conducted through an infusion into the left ventricle of the heart. After the perfusion, the brain was postfixed for 24 h in 4% PFA solution. Then, the brain was sequentially immersed in 10%, 20%, and 30% sucrose solution in PBS for three consecutive days. The fixed brain was embedded in O.C.T compound (Sakura Finetek Japan Co. Ltd., Tokyo, Japan) with hexane (cat no. 082–00426, Wako Pure Chemical Industries Ltd., Japan) and dry ice. Neonatal mice at P0.5 were anesthetized by cooling the body temperature with indirect contact with ice. Mice were then transcardially perfused with PBS, followed by perfusion with 4% PFA through infusion into the left ventricles. The skin of the head was separated from the body and the brain was collected. Thereafter, the fixed brain was postfixed for 24 h in 4% PFA and immersed for three consecutive days in 10%, 20%, and 30% sucrose solution. The brain was then embedded in O.C.T compound (Sakura Finetek Japan Co. Ltd., Tokyo, Japan).

### Sample preparation for immunohistochemistry

Embedded brains were sectioned coronally at 20 μm thickness using CryoStar NX70 cryostat (Thermo Scientific, Yokohama, Japan). The sections were placed on adhesive micro glass slides (MAS-05, Matsunami Glass Industries Ltd. Osaka, Japan) and dried for 1 h. The glass slides were dipped into cold methanol (cat no. 137–01823, Wako Pure Chemical Industries Ltd., Osaka, Japan) for 20 min and dried for 1 h. Finally, the slides were preserved at −85°C for further use. Sixteen coronal slices with 20 μm thickness were prepared for E16.5 and 24 coronal slices were prepared for E17.5. Slices were obtained starting from the line connecting both posterior edges of the orbitae. Slices situated in the middle of the collected samples (E16.5; slice numbers 7 to 10, E17.5; slice numbers 11 to 14) were used for immunohistochemistry. For P0.5 brains, 45 coronal slices with 20 μm thickness were prepared, starting from the line connecting both posterior edges of the orbitae. CC at P0.5 mostly started from the 15–17^th^ slice (confirmed in 12 out of 16 WT mice) and terminated at the 65–73^rd^ slice (confirmed in 5 WT and 1 KO). Thus, slices obtained from the anterior half of the middle portion of the CC (slice numbers 35 to 45) were used for the immunohistochemical analyses to evaluate the midline crossing of callosal axons.

### Immunohistochemistry

The glass slides with sections were dried for 30 min and washed in PBS with 0.1% polyoxyethylene (20) sorbitan monolaurate (cat no.166-21213, Wako Pure Chemical Industries Ltd., Osaka, Japan) for 15 min. The slides were washed in PBS for 15 min, heated in 10% HistoVT One (cat no. 06380–05, Nacalai Tesque Inc. Kyoto, Japan) solution for 30 min at 75°C, and left for 30 min at room temperature. The slides were then washed in PBS for 15 min and roundly marked around the sections with liquid-blocking pen (Pap pen, DAIDO SANGYO, Saitama, Japan). Sections were treated with blocking buffer containing 4% BSA (cat no. A8022, Sigma Aldrich, St. Louis, MO, USA), 4% goat or donkey serum (cat no. 325285–4, Abcam; cat no. D9663, Sigma Aldrich), 10% sodium azide (cat no. 26628-22-8, Sigma Aldrich), and 0.02% Triton-X-100 (cat no.161-0407, Bio-Rad Laboratories, Hercules, CA94547), and were preserved for 1 h at room temperature. Alternatively, sections were treated with Blocking One Histo (cat no. 06349–64, nacalai tesque, Kyoto, Japan) for 10 min at room temperature. Sections were then incubated with primary antibodies overnight at 4°C. Primary antibodies used for the detection of the target proteins during immunostaining were anti-human/mouse PlexinA1 (1:400, AF4309, R&D Systems, Minneapolis, USA), anti-mouse/rat Neuropilin-1 (1:100, AF566, R&D Systems), anti-Semaphorin3A (1:100, PAB7888, Abnova, Taipei, Taiwan), anti-SEMA3C (C-term) (1:400, SAB1304782, Sigma Aldrich), anti-L1CAM (1:200, ab24345, Abcam), anti-CR (1:100, AF5065, R&D Systems), anti-CR (1:100, ab702, Abcam), and purified mouse anti-human DCC (1:800, 554223, BD Biosciences, New Jersey, USA). The samples were washed for 50 min in PBS exchanged five times. Sections were then incubated with secondary antibodies diluted in either the blocking buffer or Signal Enhancer HIKARI for Immunostain Solution A (cat no. 02373–54, nacalai tesque) for 1 h at room temperature. Secondary antibodies used for detection of the target proteins during immunostaining were Alexa flour 555 donkey anti-goat (1:500, A-21932, Life Technology), Alexa flour 555 donkey anti-rabbit (1:500, A31572, Thermo Fisher Scientific), Alexa flour 488 donkey anti-mouse (1:200, ab150105, Abcam), Alexa flour 488 goat anti-mouse (1:200, A-11057, Thermo Fisher Scientific), Alexa flour 488 donkey anti-rat (1:500, A21208, Life Technologies), Alexa flour 488 donkey anti-rabbit (1:200, A21206, Thermo Fisher Scientific), Alexa flour 488 donkey anti-goat (1:200, A21932, Life Technologies), and Alexa flour 488 F(ab)_2_ fragment of goat anti-mouse (1:200, A11017, Thermo Fisher Scientific). Sections were washed in PBS exchanged five times for 50 min and finally covered with micro cover glasses (Matsunami Glass Industries Ltd.) using Mount-Quick Aqueous (Daido Sangyo Co., Ltd.) or Fluoro-KEEPER Antifade Reagent, Non-Hardening Type with DAPI (cat no. 12745–74, nacalai tesque). To visualize cellular nuclei of sections mounted with Mount-Quick Aqueous, bisBenzimide H 33258 (cat no. 14530, Sigma Aldrich, USA) was used. The samples were preserved overnight at 4°C and slices were imaged with an All-in-One Fluorescence Microscope (BZ-X710; Keyence, Osaka, Japan) controlled with BZ-X viewer version 1.3.1.1. Images were analyzed with BZ-X Analyzer version 1.4.0.1.

### DiI anterograde axonal tracing

Transcardial perfusion of PBS into the mice at E17.5 was used to remove blood and successive perfusion of 4% PFA was performed to fix the brain tissues. Cerebella and olfactory bulbs were removed from the fixed brains. The brains were placed in 4% PFA for 24 h for postfixation. Skull fragments and meninges were carefully removed and the postfixed brain was coronally cut at both rostral and caudal sites using a neonatal mouse brain slicer (Zivic Instruments, Pittsburgh, PA15237) to obtain a brain sample with coronal cross-section planes. The brain samples were placed on slide glasses with the rostral cross-section plane facing upward. Then, 0.03 μl of 10% DiI (Invitrogen, NY, USA) solution in N, N-dimethylformamide was injected into the cingulate cortex of the right hemisphere with a Neuros Syringe (#7000.5, Hamilton, NEVADA, USA) or a DiI crystal was placed in the cingulate cortex under an operating microscope. The brain samples treated with DiI were drenched with 4% PFA in PBS in a wet box at 35°C for two weeks. The brain samples were sectioned at 200 μm with a MicroSlicer (DOSAKA EM CO., LTD., Kyoto, Japan). The sections were attached to glass slides (Matsunami Glass Industries Ltd.), covered in Mount-Quick Aqueous (Daido Sangyo Co., Ltd.), and viewed under the BZ-X710 All-in-One Fluorescence Microscope (Keyence).

### Western blotting

The medial regions containing the cingulate gyrus, cingulate and neocortex-derived axons, and the CC at callosal axon pre-crossing (E16.5) and post-crossing (E17.5) stages were cut from the brain sample with coronal cross-section planes at both rostral and caudal sites using the neonatal mouse brain slicer (Zivic Instruments, Pittsburgh, PA15237). The dissected tissues were homogenized with T-PER Tissue Protein Extraction Reagent containing 25 mM bicine, 150 mM sodium chloride at pH 7.6 (Thermo Fisher Scientific Inc. IL, USA), cOmplete ULTRA Tablets, Mini, *EASYpack* Protease Inhibitor Cocktail (05892970001, Roche Applied Science, Penzberg, Germany), and PhosStop (4906845001, Roche applied Science). The homogenates were centrifuged at 10,000 rpm for 10 min at 4°C to obtain the supernatant. Protein concentrations in the sample were determined using the bicinchoninic acid (BCA) protein assay kit (Thermo Fisher Scientific Inc.). The protein samples were prepared for electrophoresis by adjusting the protein concentration to 2 μg/μL with sample buffer (44.5% glycerol, 0.125 M Tris-HCL, pH 6.8, 4% SDS, Bromophenol Blue (optimal), and 10% β-mercaptoethanol) and boiled for 5 min at 95°C. An equal amount of protein (10–20 μg) was loaded into each well and separated by sodium dodecyl sulfate polyacrylamide gel electrophoresis at 150 V for 60 min. The proteins were then transferred to polyvinylidene difluoride (PVDF) membranes at 100 V for 1 h. After transfer, the membranes were blocked for 1 h using the PVDF Blocking Reagent for Can Get Signal (NYPBR01, Toyobo Co. Ltd., Osaka, Japan) and incubated for 1 h at room temperature on the Labo Shaker (Model BC-730, BC Bio CRAFT, Tokyo, Japan). The membranes were washed for 30 min (3 times for 10 min each) in TBS-T (20 mM Tris Base, 150 mM NaCl, 0.05% Tween 20, PH 7.6). After washing, the membranes were incubated overnight at 4°C on the Labo Shaker with the following primary antibodies: β-Actin (1:10000, 13E5, Cell Signaling Technology); anti-mPlexinA1 (1:5000, AF4309, R & D Systems); anti-m/rNeuropilin-1 (1:20000, AF566, R& D Systems); anti-Semaphorin3A (1:10000, ab23393, Abcam); and anti-SEMA3C (C-term) (1:5000, SAB1304782, Sigma Aldrich) in Can Get Signal immunoreaction enhancer solution-1 (NKB-101, Toyobo Co. Ltd.). The membranes were washed again for 30 min (3 times for 10 min each) at room temperature with TBS-T, incubated with horseradish peroxidase-conjugated secondary antibody in Can Get Signal immunoreaction enhancer solution-2 (NKB-101, Toyobo Co. Ltd.) for 1 h at room temperature on the Labo Shaker, and then were washed again in TBS-T for 30 min (3 times for 10 min each). The membranes were reacted with ECL prime reagents (GE Healthcare, Piscataway, NJ, USA). Analyses were performed using Image Quant LAS-4000 (GE Healthcare Biosciences, Sweden).

### Statistical analysis

Statistical analysis in the present study was performed with Ekuseru-Toukei 2015 (Social Survey Research Information Co., Ltd., Tokyo, Japan), which adds a statistical analysis menu to Excel. The midline crossing of WT and PlexinA1 KO callosal axons at E17.5 and P0.5 was evaluated using the χ^2^-test. The expression levels of the target protein detected by western blotting in the WT and PlexinA1 KO midline area at E16.5 and E17.5 were compared using the student’s *t*-test. *P*<0.05 was regarded as statistically significant.

## Results

### Expression of PlexinA1 in the developing cortical midline

**Fig 1:**
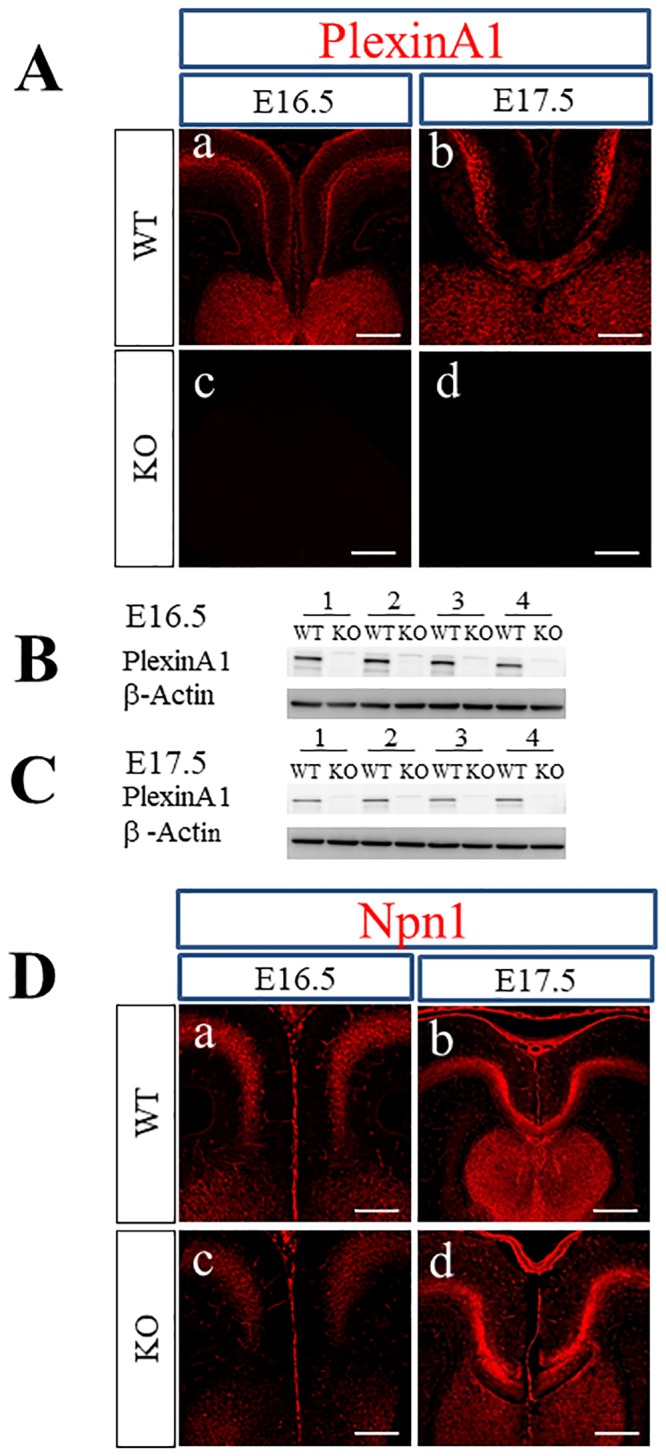
Localization of PlexinA1 and Npn1 in the coronal sections of WT and PlexinA1 KO brains at E16.5 and E17.5. (A) Immunohistochemistry with anti-PlexinA1 antibody revealed the localization of PlexinA1 only in WT brains at E16.5 and E17.5 (a, b) as well as the lack of PlexinA1 in PlexinA1 KO brains (c, d). Scale bars: 200 μm. (B, C) Western blot analysis using anti-PlexinA1 antibodies was performed with tissue lysates from the medial regions covering the cingulate cortex, axons from the cingulate and neocortex, and the CC of E16.5 and E17.5 brains. PlexinA1 protein was detected in WT brains (B; E16.5, C; E17.5) but not in PlexinA1 KO brains (B; E16.5, C; E17.5). (D) Immunohistochemistry with anti-Npn1 antibodies revealed localization of Npn1 in both WT (a; E16.5, b; E17.5) and PlexinA1 KO (c; E16.5, d; E17.5) brains at E16.5 and E17.5. Scale bars: 200 μm.

Together with Npn1 and L1CAM, PlexinA1 is expressed in cingulate pioneer neurons of C57BL6/J mice from E15 to E17 [19]. The mRNAs of class 3 Semas (Sema3s) including Sema3A and Sema3C are also expressed in the developing cortical midline of C57BL6/J mice from E15 to E17 [19]. To confirm the localization of PlexinA1, Npn1, Sema3A, and Sema3C in BALB/cAJ mice at E16.5 and E17.5, we performed expression analysis using immunohistochemical staining and western blotting. As shown in Fig 1A-a, PlexinA1 was located both superficial to and deep within the cingulate cortex as well as in the septum in WT mice at E16.5 (Fig 1A-a). At E17.5, PlexinA1 was localized deep within the cortex, with the callosal axons traversing the midline and the septum of the WT mice (Fig 1A-b; S1 Fig). In contrast, PlexinA1 expression was not detected in any areas of the PlexinA1 KO mouse brain at both E16.5 and E17.5 (Fig 1A-c and 1d; S1 Fig), confirming the specificity of the anti-PlexinA1 antibodies used. Western blotting using tissue lysates derived from the cortical midline of WT and PlexinA1 KO mice at E16.5 and E17.5 revealed PlexinA1 in WT, but not in PlexinA1 KO mice (Fig 1B and 1C). The expression of Npn1 was confirmed in the cortical midline areas of WT and PlexinA1 KO at E16.5 and E17.5. In agreement with the study reporting the localization of Npn1 in the axons of the cingulate cortex [19], Npn1 was localized to cingulate axons of both genotypes at E16.5 and E17.5 (Fig 1D). In most of the WT mice at E17.5, Npn1 was localized to the cingulate axons crossing the midline (Fig 1D-b). In contrast, Npn1+ callosal axons did not cross the midline in most PlexinA1 KO mice at E17.5 (Fig 1D-d).

**Fig 2:**
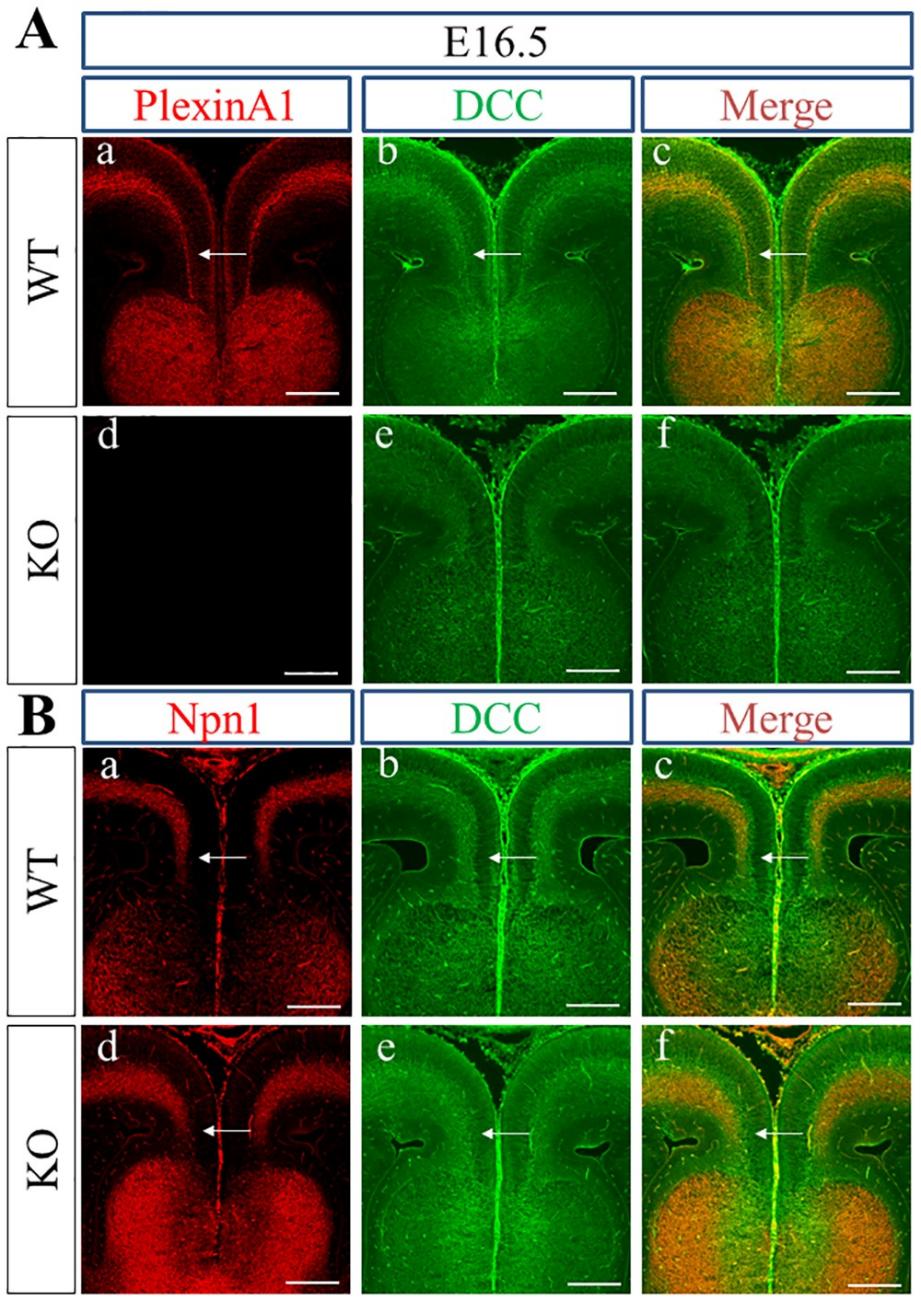
Localization of PlexinA1, DCC, and Npn1 in WT and PlexinA1 KO brains at E16.5. (A) PlexinA1 (red) was expressed in the DCC+ area (green) deep within the cingulate cortex in WT brains at E16.5 (arrows in a, b, c), but only DCC (green) was detected deep within the cingulate cortex in PlexinA1 KO brains (d, e, f). (B) Expression of Npn1 (red) and DCC (green) overlapped with the DCC+ area (green) in WT (arrows in a, b, c) and PlexinA1 KO (arrows in d, e, f) mouse brains at E16.5. Scale bars: 200 μm.

**Fig 3:**
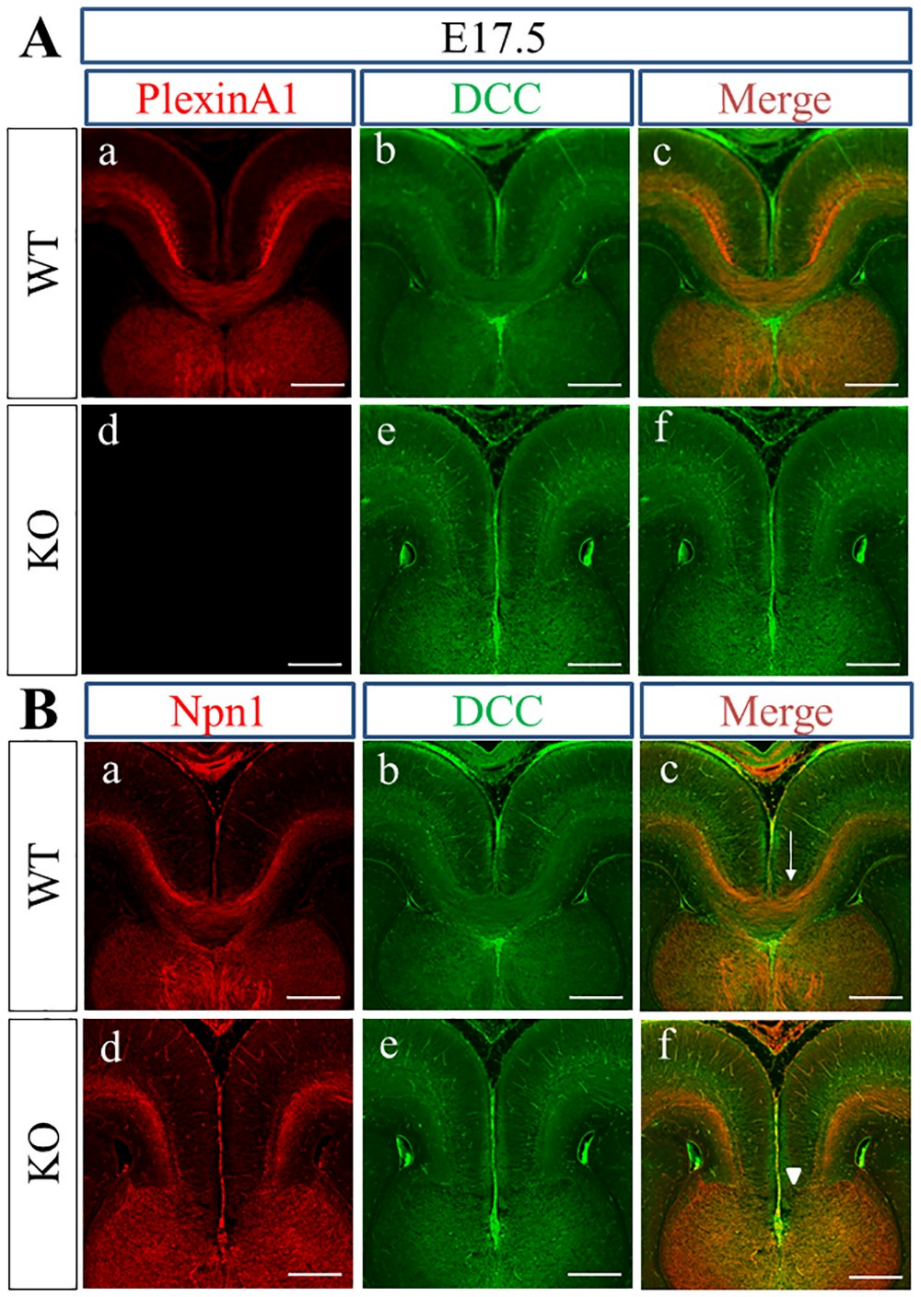
Localization of PlexinA1, DCC, and Npn1 in WT and PlexinA1 KO brains at E17.5. (A) At E17.5, PlexinA1 is expressed alongside DCC in cingulate axons and neocortical callosal axons crossing the midline in WT mice brains (c), whereas only DCC is detected in PlexinA1 KO brains (f). (B) Expression of Npn1 (red) is overlapped with the dorsal side of the DCC+ area (green) in WT (a, b, c) and PlexinA1 KO (d, e, f) mice brains at E17.5. Callosal axons fail to cross the midline (arrowhead in f) in PlexinA1 KO brain sections but do cross the midline in WT brain sections (arrow in c). Scale bars: 200 μm.

Next, we tried to determine whether PlexinA1 is localized to Npn1+ pioneer axons in cingulate-derived callosal axons. Because both anti-PlexinA1 and anti-Npn1 antibodies were polyclonal goat IgG, we could not use these antibodies together for double immunofluorescence study. Thus, we first performed double immunofluorescence staining of PlexinA1 and deleted in colorectal cancer (DCC) as a substitute for Npn1 in WT and PlexinA1 KO mice at E16.5 to E17.5. DCC has been shown to be expressed in cingulate-derived axons [54]. At E16.5, PlexinA1 was colocalized with the DCC+ area in WT brains (Fig 2A). In the double immunofluorescence staining of Npn1 and DCC, the localization of Npn1 nearly overlapped with that of DCC in WT brain at E16.5 (Fig 2B). Thus, PlexinA1 was colocalized with Npn1+ cingulate-derived callosal axons in WT mice at E16.5. In WT mice at E17.5, PlexinA1 was colocalized with the DCC+ area deep within the cingulate cortex and with axons derived from the cingulate and neocortex crossing the midline (Fig 3A). Npn1 was colocalized with the dorsal side of the DCC+ area in cingulate-derived axons in WT and PlexinA1 KO brains at E17.5 (Fig 3B). Taken together, the localization of PlexinA1 was identified in the Npn1, DCC double+ cingulate, and DCC+ callosal axons at E17.5 in BALB/cAJ mice.

### Expression of ligands for PlexinA1 in the developing cortical midline

**Fig 4:**
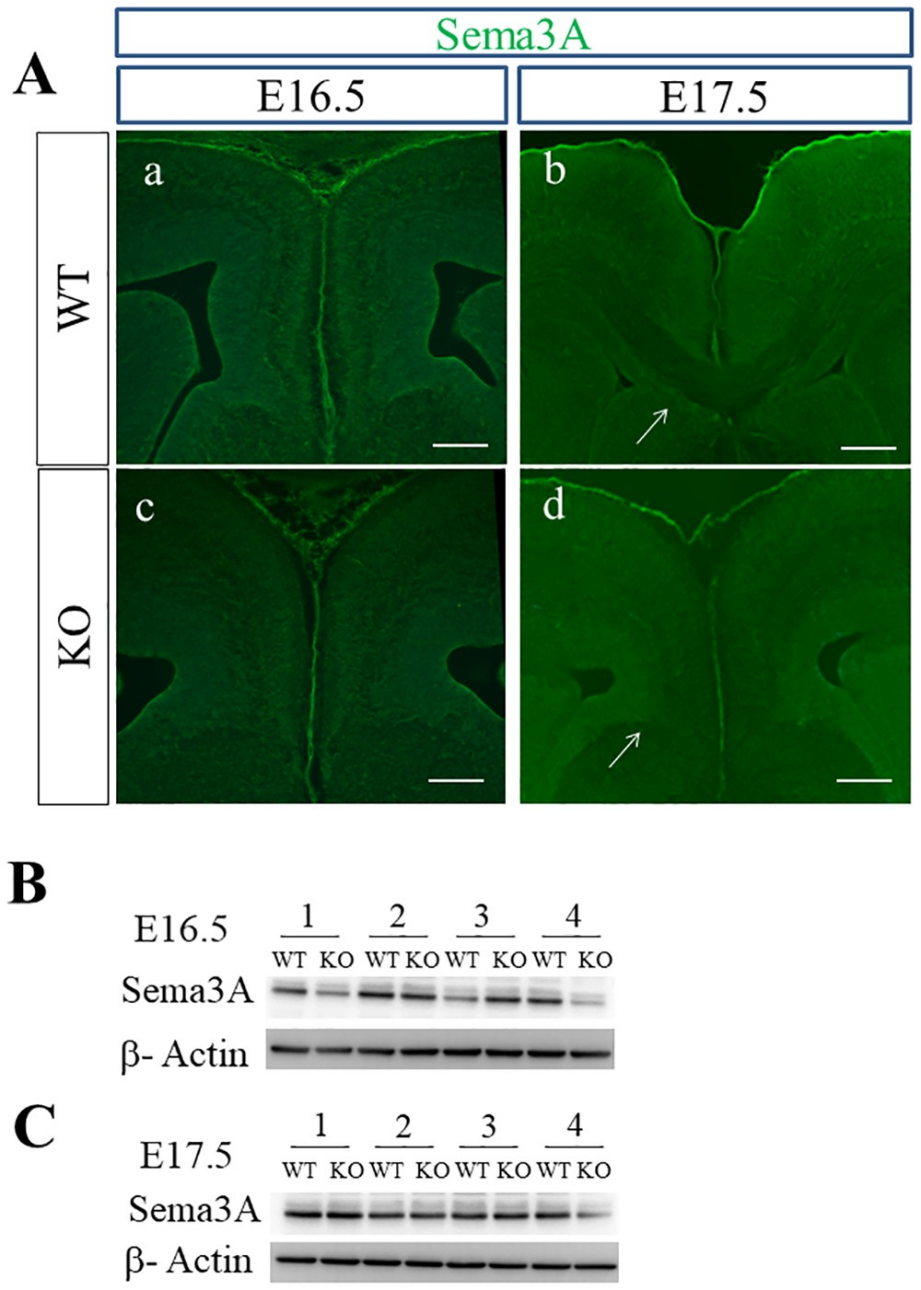
Expression of Sema3A in the cortical midline of WT and PlexinA1 KO brains at E16.5 and E17.5. (A) Immunohistochemistry confirmed the expression of Sema3A in the cingulate cortex of brains of WT and PlexinA1 KO mice at E16.5 and E17.5. At E17.5, Sema3A was also expressed in the subcallosal sling in both genotypes (arrows in b, d). Scale bars: 200 μm. (B, C) Western blotting revealed Sema3A in tissue lysates in the cortical midline of both genotypes at E16.5 (B) and at E17.5 (C).

Expression of Sema3A, one of the ligands for Sema3 holoreceptors, could be confirmed in the cortical midline of the brain of WT and PlexinA1 KO mice using immunohistochemical staining and western blotting. The expression of Sema3A was detected in the cingulate cortex of brains of WT and PlexinA1 KO mice at E16.5 (Fig 4A-a and 4c). In WT and PlexinA1 KO brains at E17.5, Sema3A was expressed in the guidepost structure known as the subcallosal sling, a band-like structure below the CC, additional to the cingulate cortex (Fig 4A-b and 4d). In the western blotting using tissue lysates derived from the cortical midline of WT and PlexinA1 KO mice at E16.5 and E17.5 (Fig 4B and 4C), there were no significant differences in the expression levels of Sema3A between both genotypes at either E16.5 or E17.5 (*p* > 0.05, Student’s t-test; Sema3A signal normalized with β-Actin from WT: n = 4 and KO: n = 4).

**Fig 5:**
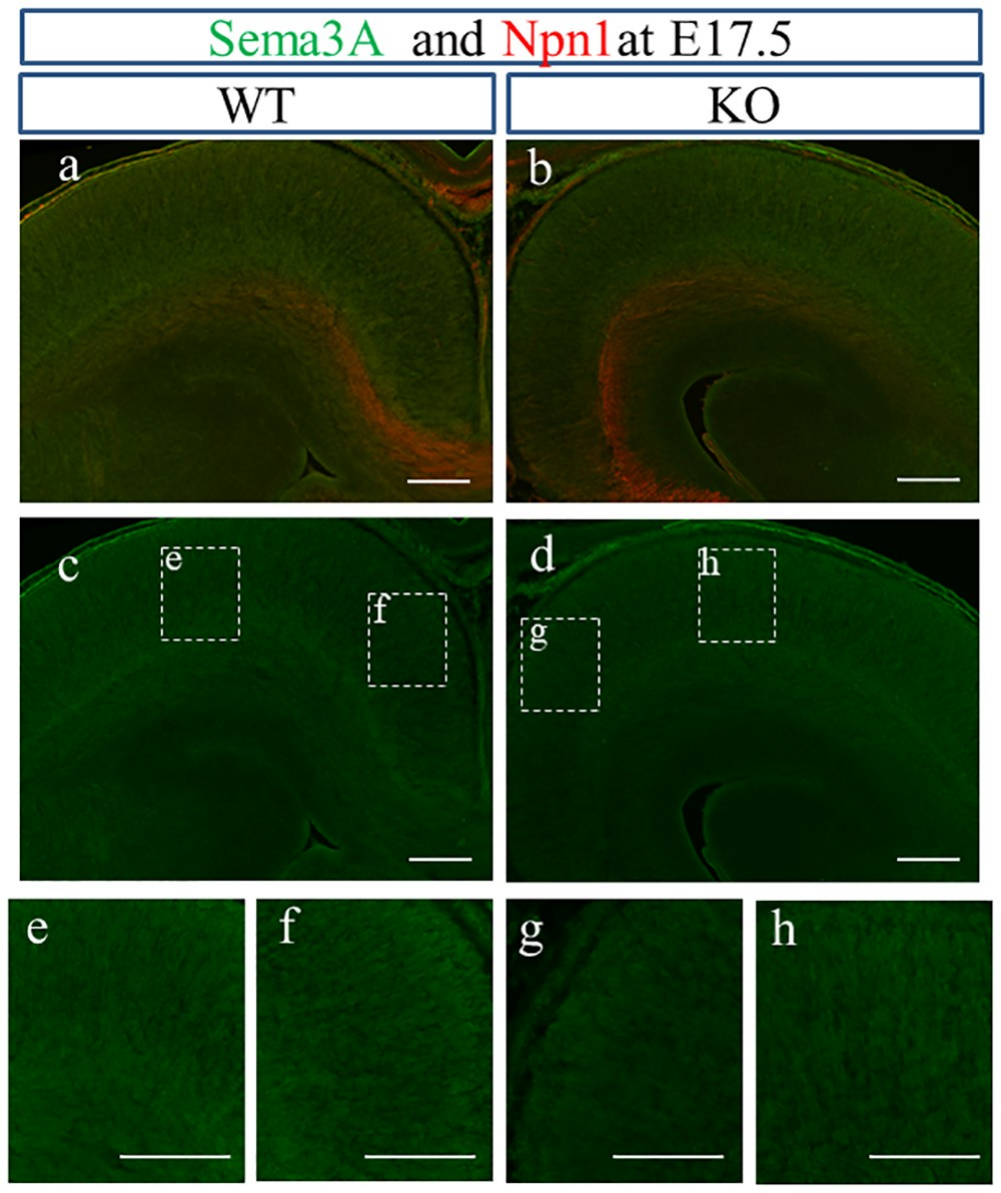
Expression of Sema3A along medio-lateral axis in the developing neocortex. The gradient of Sema3A expression from lateral-high to medial-low is observed in the neocortex of WT (a; gradient from e to f) and KO (b; gradient from h to g). (E) Npn1 showed a gradient of expression with lateral-low to medial-high in the neocortex of both genotypes (a, b). e: lateral, f: medial, g: medial, h: lateral in lower stand: higher magnification of inset of c and d. Scale bars: 200 μm.

Sema3A mRNA is expressed in the developing neocortex following the gradient from lateral-high to medial-low, and Npn1, the receptor of Sema3A is expressed on callosal axons extending from upper layer neurons [55]. Because Sema3A expressed ectopically suppresses the extension of callosal axons orienting medially, the gradient of Sema3A expression in the developing neocortex may be a mechanism in which callosal axons initially extend toward the midline from the developing neocortex [55]. To ask if the gradient of Sema3A exists in the developing neocortex of WT and KO mice under BALB/cAJ background, we analyzed the expression of Seam3A and Npn1 along the mediolateral axis of developing neocortex on E15.5–17.5. Sema3A tended to be expressed in the gradient from lateral-high to medial-low in the neocortex positioned distantly from the midline of both genotypes on E17.5 (Fig 5). In contrast, Npn1 was expressed with the opposite gradient from lateral-low to medial-high in the brains of both genotypes on E17.5 (Fig 5). The specificity of the antibody against Sema3A was confirmed by positive staining in the hippocampus (S2 Fig), which was similar to the pattern shown in another report [56].

**Fig 6:**
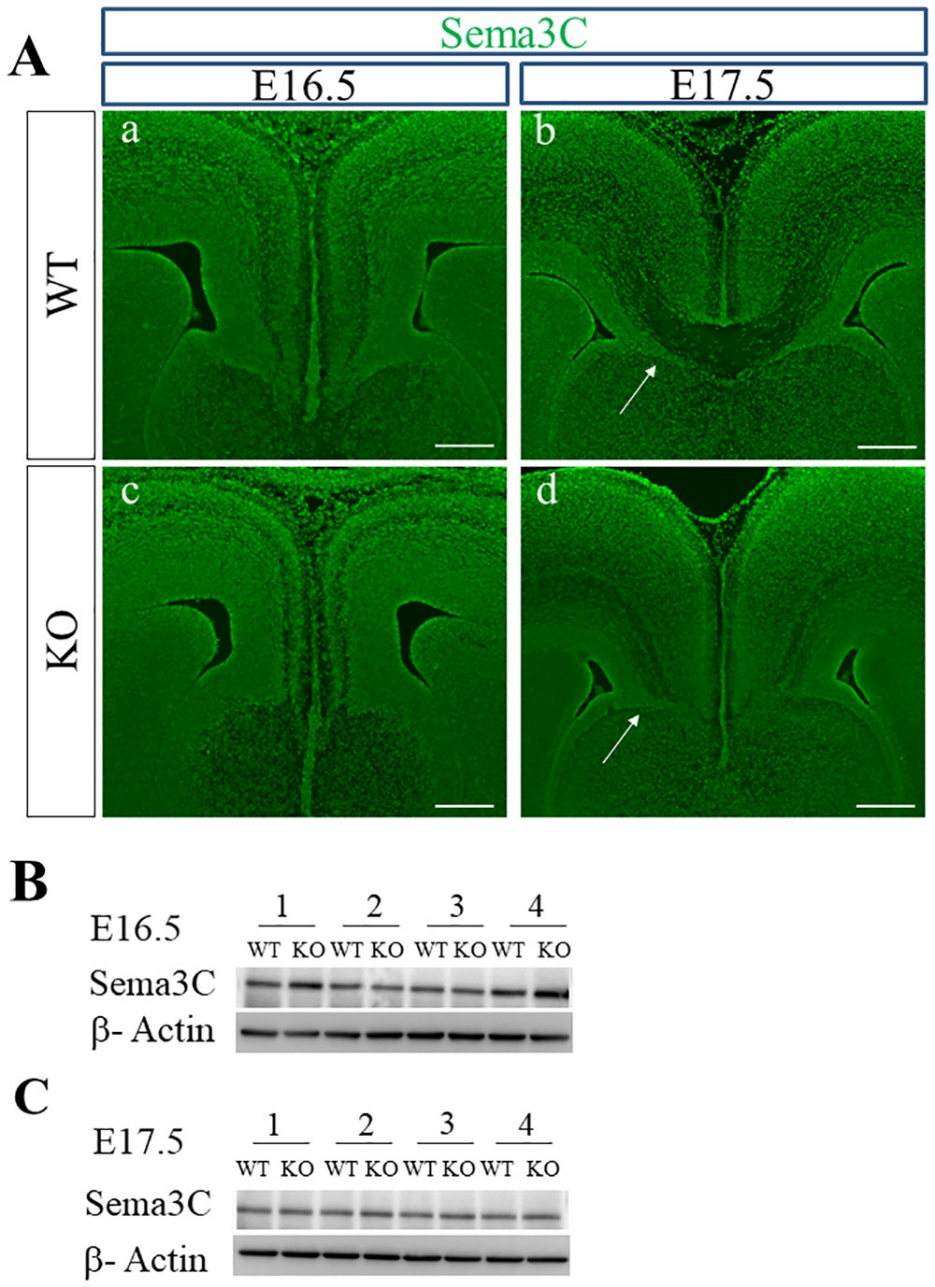
Expression of Sema3C in the cortical midline of WT and PlexinA1 KO brains at E16.5 and E17.5. (A) Immunohistochemistry confirmed the expression of Sema3C in the cingulate cortex of brains of WT and PlexinA1 KO mice at E16.5 and E17.5. At E17.5, Sema3C was also expressed in the indusium griseum (in b and d) and the subcallosal sling (arrows in b and d) in both genotypes. Scale bars: 200 μm. (B, C) Western blotting detected Sema3C in tissue lysates in the cortical midline of both genotypes at E16.5 (B) and at E17.5 (C).

Expression of Sema3C, another ligand for Sema3 holoreceptors with chemoattractive activity, could be also confirmed in the cortical midline of WT and PlexinA1 KO brains by immunohistochemical staining and western blotting. Sema3C was expressed in the cingulate cortex of WT and PlexinA1 KO brains at E16.5 (Fig 6A-a and 6c). In WT and PlexinA1 KO brains at E17.5, Sema3C was expressed in guidepost structures including the indusium griseum and subcallosal sling additional to in the cingulate cortex (Fig 6A-b and 6d). In the western blotting using tissue lysates derived from the cortical midline of WT and PlexinA1 KO mice at E16.5 and E17.5 (Fig 6B and 6C), there were no significant differences in the expression levels of Sema3C between both genotypes at either E16.5 or E17.5 (*p* > 0.05, Student’s t-test; Sema3C signal normalized with β-Actin from WT: n = 4 and KO: n = 4). The specificity of the antibody against Sema3C was confirmed by positive staining in the intermediate zone (IMZ) and diffuse staining in the cortical plate and subventricular zone (S3 Fig), which supported the results of the article reporting on the expression of Sema3C mRNA in the developing neocortex [57].

**Fig 7:**
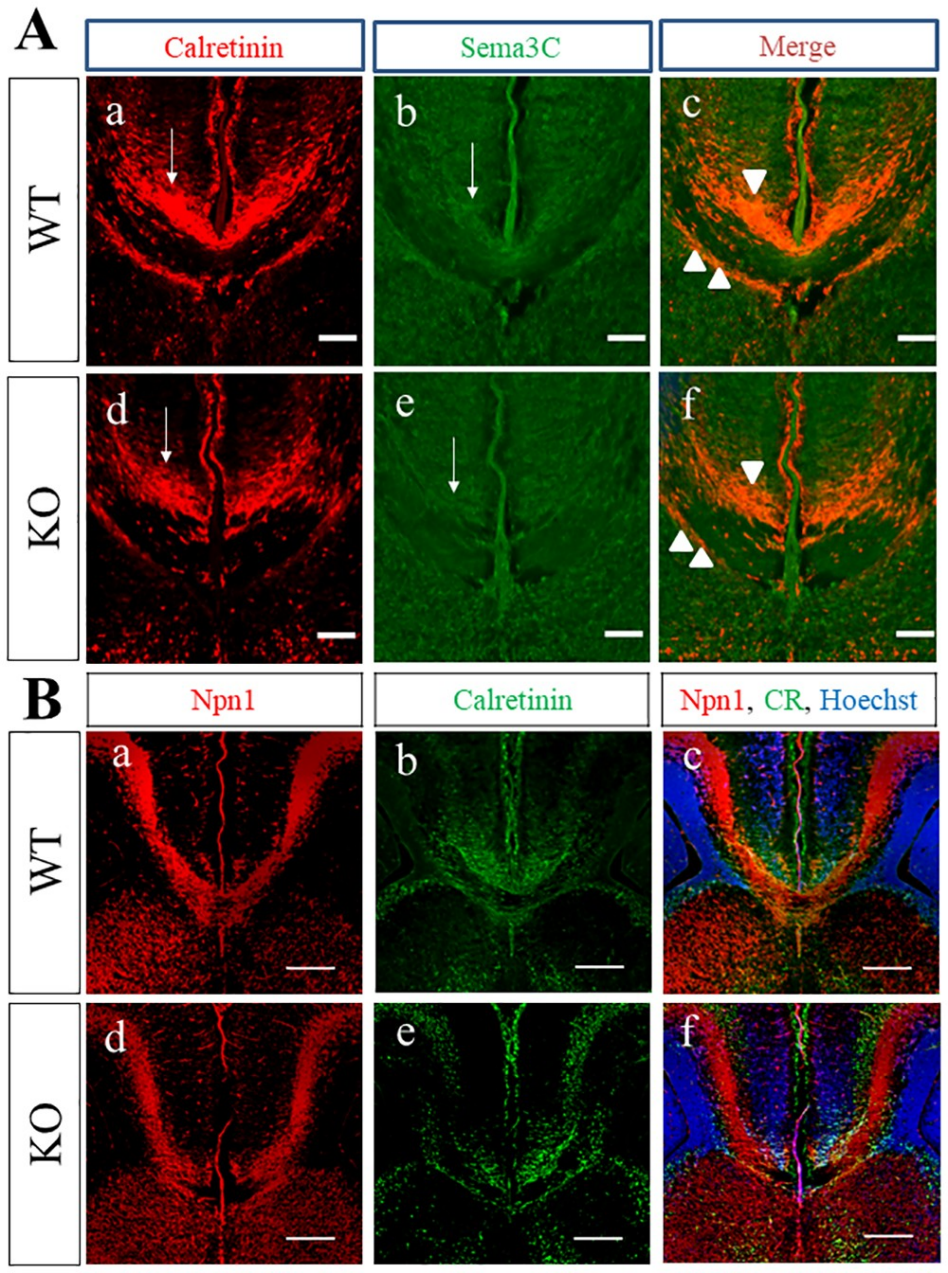
Localization of CR, Sema3C, and Npn1 in the coronal brain sections of WT and PlexinA1 KO mice at E17.5. (A) Immunohistochemistry revealed the expression of Sema3C (arrows in b and e) in the CR-positive cells (arrows in a and d) in the cortical midline of brains of WT and PlexinA1 KO mice at E17.5. Sema3C in CR-double positive cells (arrows) were observed in the indusium griseum (arrowhead) and cingulate cortex, in the middle of the white matter of the CC and in the subcallosal sling (double arrowhead) in both genotypes at E17.5 (c, f). Scale bars: 200 μm. (B) Npn1+ callosal axons extending toward the CR+ cells were found in the midline of both WT (c) and PlexinA1 KO mice (f) at E17.5. Npn1+ callosal axons crossed the midline in WT mice (a) but not in PlexinA1 KO mice at E17.5 (d). Scale bars: 200 μm.

CR+ glutamatergic neurons, which transiently emerge at the cortical midline, have been proposed to attract the extension of callosal axons to the midline by secreting Sema3C during CC development [25]. CR expression was confirmed at the cortical midline of WT and PlexinA1 KO brains at E17.5. CR was expressed in the indusium griseum, subcallosal sling, and the IMZ in both genotypes with similar expression patterns (Fig 7A-a and 7d). Double immunofluorescence staining of CR and Sema3C showed the overlap of CR and Sema3C in the indusium griseum, subcallosal sling, and the IMZ in both genotypes (Fig 7A-c and 7f), supporting a previous report of CR+ cells at the cortical midline as the guideposts secreting Sema3C [25]. To examine the extension of Npn1+ callosal axons toward the CR+ guideposts during CC development, we performed double immunofluorescence staining of Npn1 and CR in WT and PlexinA1 KO brains at E17.5. The results showed that Npn1+ callosal axons in both genotypes extended toward the CR+ cells residing at the midline (Fig 7B-c and 7f). Npn1+ callosal axons in WT mice crossed the midline but those in PlexinA1 KO mice stalled just anterior to the midline (Fig 7B-a and 7d).

### Midline crossing of Npn1+ callosal axons was impaired in PlexinA1 KO mice at E17.5

**Fig 8:**
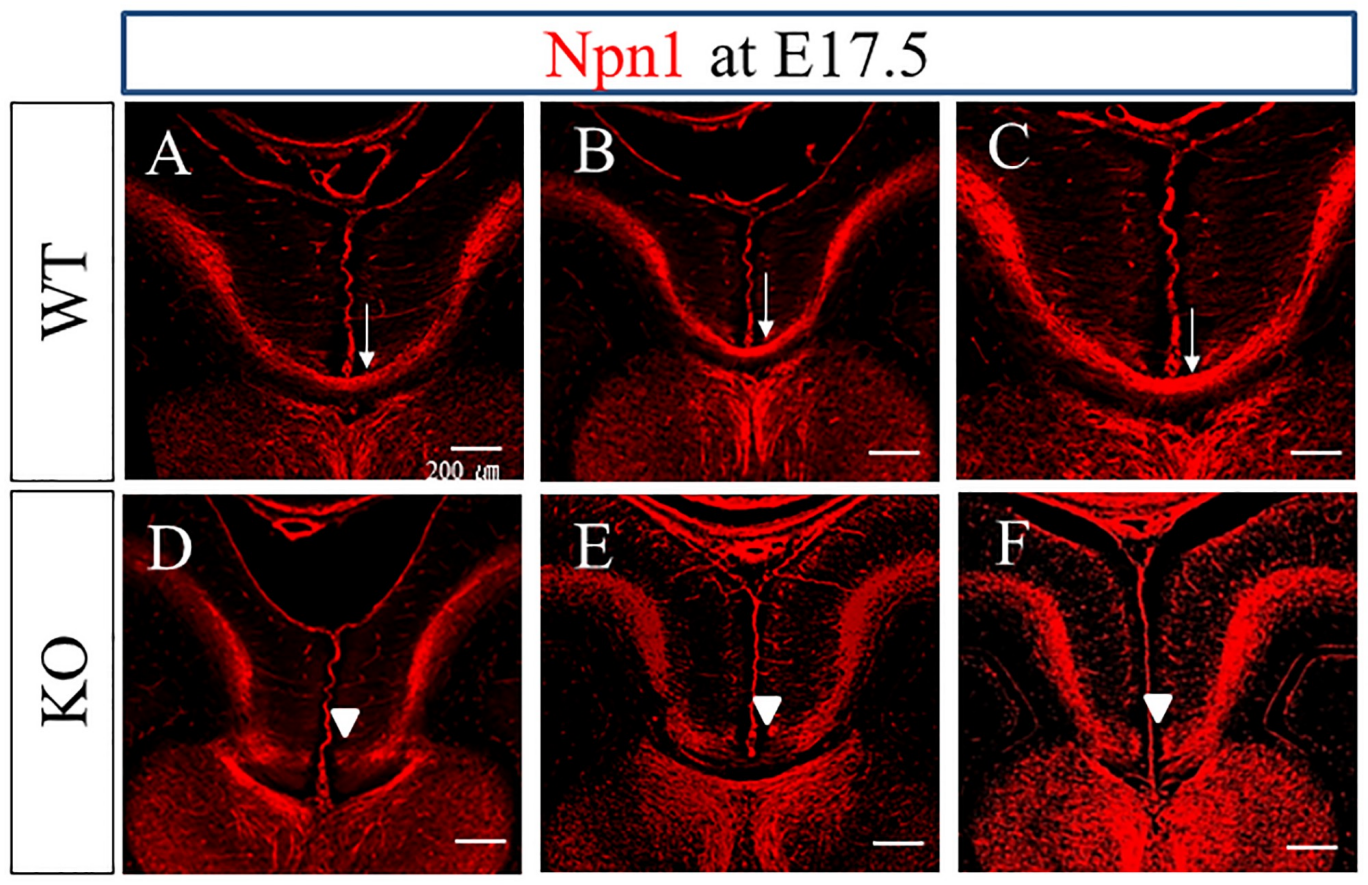
Comparison of the CC Phenotypes in WT and PlexinA1 KO brains at E17.5. Npn1+ callosal axons crossed the midline in 18 out of 24 WT mice at E17.5 (arrows in A, B, and C). In contrast, Npn1+ callosal axons crossed the midline only in four out of 25 PlexinA1 KO mice at E17.5 and did not cross the midline in 21 KO mice (arrowheads in D, E, and F). Scale bars: 200 μm.

We analyzed the extension and the midline crossing of Npn1+ callosal axons in WT and PlexinA1 KO brains at E17.5 with Npn1 immunohistochemical staining (Fig 8). Midline crossing was confirmed in 18 out of 24 WT mice at E17.5, and was not detected in the other six WT mice. In contrast, midline crossing was confirmed in four out of 25 PlexinA1 KO mice, and was not detected in 21 KO mice. Thus, the incidence of midline crossing of Npn1+ callosal axons was significantly lower in PlexinA1 KO mice at E17.5 than that in WT mice at E17.5 (χ^2^ test, *P* < 0.05, S1 Table). We observed the stalled end of Npn1+ callosal axons just anterior to the midline in most of the PlexinA1 KO mice without midline crossing at E17.5 (Fig 8D, 8E and 8F; 15 out of 21 KO). Results obtained from tracing the tract through the injection of DiI into the cingulate cortex of brain sections from WT and PlexinA1 KO mice at E17.5 also supported the immunohistochemical staining results for Npn1 (S4 Fig). Midline crossing was confirmed in nine out of 10 WT mice. In contrast, midline crossing was confirmed in only two out of 16 PlexinA1 KO mice, and was not detected in the other 14 KO mice. Thus, the incidence of midline crossing of DiI-labeled callosal axons was significantly lower in PlexinA1 KO mice at E17.5 than that in WT mice (χ^2^ test, *P* < 0.05, S2 Table). We did not detect mistargeting to any ectopic areas other than the midline in the DiI-labeled callosal axons of PlexinA1 KO mice at E17.5.

### PlexinA1 KO Mice at P0.5 display AgCC

**Fig 9:**
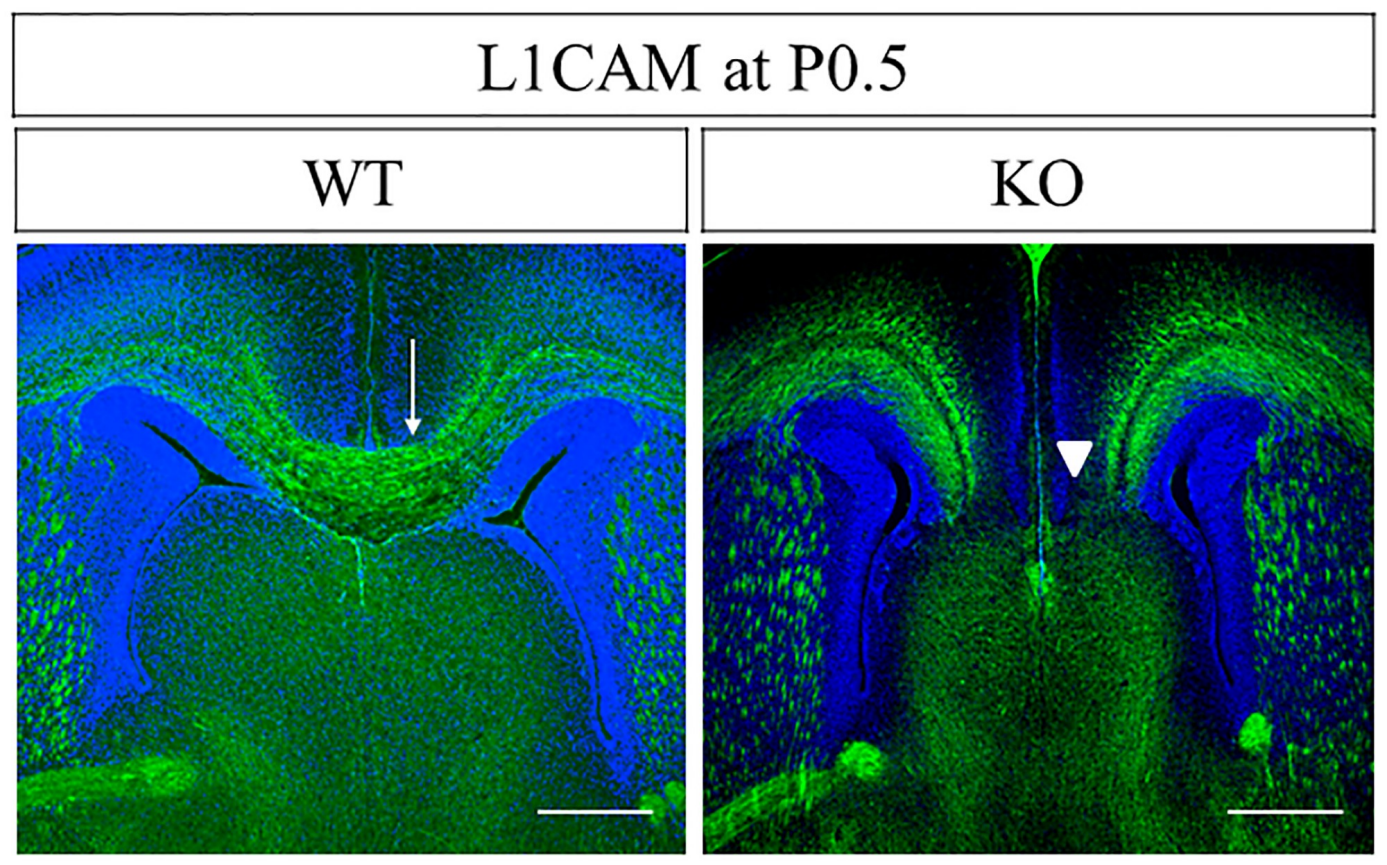
Agenesis of CC in PlexinA1 KO mice at P0.5. Coronal brain sections of WT and PlexinA1 KO mice at P0.5 were stained with anti-L1CAM antibody in the middle levels of CC. L1CAM+ callosal axons crossed the cortical midline in 16 out of 16 WT mice (arrow in WT). In contrast, L1CAM+ callosal axons did not cross the midline in 10 out of 13 PlexinA1 KO mice (arrowheads in KO). Scale bars: 200 μm.

To examine how the stalled state anterior to the midline of PlexinA1-deficient callosal axons at E17.5 changes at P0.5 approximately two days later, we visualized the callosal axons of WT and PlexinA1 KO mice at P0.5 with immunohistochemical staining using antibodies against L1CAM. L1CAM+ callosal axons crossed the midline in 16 out of 16 WT mice, but not in 10 out of 13 PlexinA1 KO mice at P0.5 (Fig 9). Thus, the incidence of midline crossing of callosal axons was significantly lower in PlexinA1 KO mice at P0.5 than that in WT mice (χ^2^ test, *P* < 0.05, S3 Table). Furthermore, in 10 out of 13 PlexinA1 KO mice, AgCC was detected in the anterior half of the CC. In contrast, AgCC was not detected in either of the 16 WT mice. The incidence of AgCC in the anterior half of the CC was significantly higher in PlexinA1 KO mice at P0.5 than in WT mice (χ^2^ test, *P* < 0.05, S4 Table).

## Discussion

The present study using PlexinA1 KO mice with a BALB/cAJ background uncovered three crucial findings regarding the guidance of callosal axons to the midline during CC development. The results showed that: 1) PlexinA1 was expressed in cingulate and neocortex-derived callosal axons at E17.5 during CC development; 2) the midline crossing of callosal axons was significantly impaired in PlexinA1 KO mice at E17.5 as compared with that in WT mice; and 3) AgCC in the anterior half of CC occurred in most of the PlexinA1 KO mice at P0.5.

The expression of PlexinA1 in axons derived from the cingulate and neocortex at embryonic stages during CC development in BALB/cAJ mice was confirmed by the combination of double immunofluorescence staining using anti-PlexinA1 and anti-DCC antibodies and double immunofluorescence staining with anti-Npn1 and anti-DCC antibodies. PlexinA1 was colocalized to Npn1+ cingulate-derived axons at E16.5 (Fig 2A and 2B). Npn1+ callosal axons had yet to cross the midline at E16.5 in BALB/cAJ WT mice and thus were still at the pre-crossing stage. In contrast to spinal commissural axons [58], PlexinA1 expression in axons and growth cones may not be lowered in Npn1+ callosal axons at the pre-crossing stage. Thus, PlexinA1 may function as a component of Sema3 holoreceptors that guide Npn1+ callosal axons to the cortical midline during CC development of BALB/cAJ mice. Accordingly, PlexinA1 together with other plexins like PlexinA3 and PlexinD1 or L1CAM [51] may be involved in the Sema3C and Npn1-mediated attraction of cingulate axons to the midline during CC development. The colocalization of PlexinA1 and Npn1 in cingulate axons is most evident in cingulate axons crossing the midline at E17.5 (Fig 3). The finding of the colocalization of PlexinA1 and Npn1 in cingulate axons of BALB/cAJ mice is consistent with the previous report indicating the expression of PlexinA1 and Npn1 in cingulate axons during CC development in C57BL/6J mice [19]. The expression of PlexinA1 was also detected in the neocortex-derived callosal axons traversing the cortical midline at E17.5 (Fig 3). Thus, PlexinA1 may have a crucial role in the guidance of cingulate and neocortex-derived callosal axons at the midline crossing and post-crossing stages as a component of Sema3 holoreceptors composed of neuropilins and plexins.

The gradient of Sema3A expression from lateral-high to medial-low in the developing neocortex was observed in the region from developing neocortex to the midline of both WT and PlexinA1 KO mice, and Npn1+ axons extended medially from the lateral side in the brains of both genotypes (Fig 5). Previous studies have revealed the crucial roles of the Sema3A/Npn1 signal in the extension of callosal axons from the neocortex and axonal positioning in the CC during CC development [55, 59]. When axons of callosal projection neurons in the developing cortex initially begin to extend toward the midline, the gradient from high to low concentration of Sema3A may orient axons from the lateral to medial direction [55]. As discussed in their work [55], callosal axons may come close to the midline following another mechanism including local guidance from several guideposts in addition to the Sema3A gradient. The Sema3A/Npn1 signal in the cortical midline operates for the axonal positioning within CC, in which axons derived from the cortex near the midline are located dorsally and those from the neocortex located far from the midline are positioned ventrally in the CC [59]. Several studies reported on callosal phenotypes related to PlexinA1 by investigating the association of PlexinA1 and its interacting molecules with CC development [26, 60, 61]. Wu et al. found the defasciculation of callosal fibers and mistargeting of callosal axons to different regions in the contralateral hemisphere by suppressing the function of PlexinA1 in callosal projection neurons of the rat somatosensory cortex (S1, S2) through in utero electroporation [26]. The study showed that Sema3A induced endocytosis in the growth cone of callosal axons during CC development and the Sema3A/Npn1 and PlexinA1-mediated signals are crucial for fasciculation of callosal fibers and correct topographical targeting of callosal fibers to homotopic regions of the contralateral hemisphere after crossing the midline of callosal axons [26]. Another study found defasciculation of callosal axons in the CC of KO mice deficient of Syb2, a synaptic vesicle protein which associates with PlexinA1 [60]. Thus, Sema3A signal through Npn1, PlexinA1, and Syb2 is crucial for fasciculation of callosal fibers by promoting Syb2-dependent vesicular transport [60]. Son et al. identified PlexinA1 as a protein with peak expression at postnatal day 7 (P7) in their proteome profiling to search molecules involved in postnatal CC development such as axon refinement [61]. To test if their molecular profiling is effective for the identification of molecules involved in the postnatal CC refinement, they examined the callosal phenotype of mice at P15 by inducing knockdown or overexpression of PlexinA1 in callosal projection neurons in the embryonic neocortex. More axons with PlexinA1 knockdown appeared to be contained in the postnatal CC compared with axons with control vectors, and axons with overexpressed PlexinA1 tended to decrease in the postnatal CC [61]. As in their discussion, PlexinA1 may be involved in the regulation of axon numbers by pruning excessive axons within CC around P7 [61]. Thus, it is conceivable that the number of axons with knockdown of PlexinA1 increases and that with overexpressed PlexinA1 decreases in the postnatal stage of CC refinement.

Previous studies have shown the crucial roles of PlexinA1 in various processes such as fasciculation of callosal fibers, homotopic projection of callosal axons to contralateral hemisphere, and axon refinement in postnatal CC [26, 60, 61]. In our study, the midline crossing rate of callosal axons was significantly lower in PlexinA1 KO mice at E17.5 compared with midline crossing rate of callosal axons in WT mice, as determined by tracing the Npn1+ callosal axons using immunohistochemistry (Fig 8, S1 Table) and DiI anterograde tracing of callosal axons (S4 Fig., S2 Table). Impaired midline crossing of callosal axons has not yet been reported in PlexinA1 KO mice. Thus, the present study reports a callosal phenotype with a midline crossing defect of callosal axons in PlexinA1 KO mice. Sema3A and Sema3C were appropriately localized in the guideposts in the cortical midline in both WT and PlexinA1 KO mice at E16.5 and E17.5. The expression level of these ligands in the PlexinA1 KO midline was equivalent to that in the WT midline at E16.5 and E17.5 (Figs 4 and 6). Thus, the PlexinA1-mediated signaling defect in the cingulate and callosal axons may be the primary cause of the midline crossing defect in PlexinA1 KO mice at E17.5. Severe misguidance of cingulate pioneering axons toward the septum and callosal defect was reported in *Npn1*^*Sema-*^ knock-in mice expressing Npn1 that was unable to bind Semas [19, 62], and specific KO mice of *Nrp1* in the cingulate cortex [16]. Since most class 3 Semas (Sema3s) bind to Nrp1 expressed on cingulate axons and properly guide cingulate axons toward the midline, loss of the Nrp1 functional response to Sema3s may cause severe misguidance of cingulate axons in these *Nrp1* mutant mice. In contrast, mistargeting of cingulate axons to the septum was not detected in the PlexinA1 KO mice at E17.5. With the DiI anterograde axonal tracing and immunohistochemistry we performed, we could not detect the mistargeting of cingulate axons toward the septum in the PlexinA1 KO mice at E17.5. The extension of cingulate and callosal axons toward the midline in PlexinA1 KO mice may proceed to a location just anterior to the midline through the function of Sema3 holoreceptors containing Npn1 and other components like PlexinA3, PlexinD1, or L1CAM [19, 25, 49, 50]. Npn1+ callosal axons were stalled just anterior to the midline at E17.5 (15 of 21 KO mice) and could stay stalled for at least 48 h until P0.5, since the midline crossing defect was observed in most of the PlexinA1 KO mice at P0.5 (S3 Table). Since the stalling of callosal axons just anterior to the midline in PlexinA1 KO mice at E17.5 implies the inability of callosal axons to traverse the midline plane, PlexinA1 on the axonal front may have the crucial role of responding to repulsive ligands such as Sema3A [19, 36, 37] when the cingulate axons begin to cross the cortical midline and enter the contralateral hemisphere. Thus, the present study reveals the potential role of PlexinA1 in axonal crossing at the cortical midline and axonal projection into the contralateral hemisphere after the appearance of cingulate and neocortical axons near the midline. To promote a better understanding of the role of PlexinA1 in CC development, further research making use of various approaches will be required to clarify if the callosal phenotype of PlexinA1 KO mice in the present study is directly owing to the lack of PlexinA1 in cingulate-derived pioneer axons or other causes such as dysplastic guideposts and midline fusion defect [63].

Our study confirmed a significantly higher incidence of AgCC in the anterior half of CC in PlexinA1 KO mice at P0.5 (Fig 9, S3 and S4 Tables). We observed the callosal phenotype in PlexinA1 KO mice with a BALB/cAJ background. The genetic background of the mouse strain is known to affect the severity of the callosal phenotype in several mutant mice [16, 64, 65, 66, 67]. Modifier genes may be responsible for the differences in the callosal phenotype in mutant mice of the same gene with a different genetic background [16]. Several substrains of BALB/c mice display callosal dysgenesis at a relatively high frequency [68, 69, 70, 71, 72]. Previous studies have reported that AgCC did not develop in 59 WT offspring derived from heterozygous transgenic mice with a BALB/cAJ genetic background [10]. In our study, AgCC was not detected in 16 WT offspring derived from heterozygous PlexinA1 KO mice with a BALB/cAJ genetic background that were examined at P0.5. Thus, the midline crossing defect of callosal axons observed in PlexinA1 KO mice is because of the absence of PlexinA1, even though the BALB/cAJ genetic background may modify the callosal phenotype.

## Conclusions

Considering the genetic background of BALB/cAJ mice, the midline crossing of callosal axons was significantly impaired in PlexinA1 KO mice at E17.5, and the incidence of AgCC in the anterior half of CC was significantly higher in PlexinA1 KO mice at P0.5 compared with the incidence of AgCC in the anterior half of CC in WT mice.

## Supporting information

S1 Fig PlexinA1 expression in the anterior and posterior part of the brain at E17.5. PlexinA1 is expressed in the anterior and posterior part of the WT brain at E17.5. scale bar: 200μm.
(TIF)

S2 Fig Sema3A expression in hippocampus at E17.5. The antibody against Sema3A showed the positive signal in the pial side of the hippocampal plate and inner marginal zone at E17.5. hp: hippocampal plate, imz: inner marginal zone, omz: outer marginal zone, DG: dentate gyrus, scale bar: 200μm.
(TIF)

S3 Fig Sema3C expression in the developing neocortex. Immunohistochemistry performed with the use of the antibody against Sema3C showed a positive signal in the cortical plate, intermediate zone, and subventricular zone in the developing neocortex on E17.5 (b: higher magnification of a). sacle bar: 200μm.
(TIF)

S4 Fig DiI anterograde axonal tracing in WT and PlexinA1 KO brain sections at E17.5. DiI was injected into the cingulate cortex of the right hemisphere of brain sections of WT and PlexinA1 KO mice at E17.5. The images were captured under optical (A and C) and fluorescent (B and D) microscopy. The bundles of callosal axons cross the midline in the contralateral hemisphere of the cerebral cortex in nine out of 10 WT mice (arrows in A and B). In contrast, the callosal axons do not cross the midline in 14 out of 16 PlexinA1 KO mice (arrow heads in C and D). Scale bars: 200 μm.
(TIF)

S1 Table Midline crossing of Npn1+ callosal axons in WT and PlexinA1 KO brain sections at E17.5. In WT mice, the midline crossing of Npn1+ callosal axons is observed in 18 out of 24 mice (75%), and is not detected in six out of 24 mice. In PlexinA1 KO mice, the midline crossing of Npn1+ callosal axons is observed in four out of 25 mice (16.6%), and is not detected in 21 out of 24 mice. The incidence of the midline crossing is significantly lower in PlexinA1 KO mice as compared with that in WT mice (χ^2^ test, *P* < 0.05).
(TIF)

S2 Table DiI tract tracing of callosal axons at E17.5. In WT, DiI-labeled callosal axons cross the midline in nine out of 10 mice (90%). In PlexinA1 KO mice, DiI-labeled callosal axons cross the midline in two out of 16 mice (12.5%). The midline crossing incidence is significantly lower in PlexinA1 KO mice as compared with that in WT mice (χ^2^ test, *P <* 0.05).
(TIF)

S3 Table Midline crossing of L1CAM+ callosal axons at P0.5. In WT, L1CAM+ callosal axons cross the midline in 16 out of 16 mice (100%). In PlexinA1 KO mice, L1CAM+ callosal axons cross the midline in three out of 13 mice (23%). The midline crossing incidence is significantly lower in PlexinA1 KO mice as compared with midline crossing incidence in WT (χ^2^ test, *P* < 0.05).
(TIF)

S4 Table Phenotype of corpus callosum in WT and PlexinA1 KO mice at P0.5. Sixteen out of 16 WT mice have normal corpus callosum (CC). In 10 out of 13 PlexinA1 KO mice, agenesis of corpus callosum (AgCC) was detected in the anterior half of the CC. +: callosal axons cross the midline. -: callosal axons do not cross the midline. CC: corpus callosum. AgCC: agenesis of corpus callosum. *χ^2^ test, *P* < 0.05.
(TIF)
