# Supplementary figures and images for: PlexinA1 is crucial for the midline crossing of callosal axons during corpus callosum development in BALB/cAJ mice
Source: PLoS One. 2019 Aug 20;14(8):e0221440. doi: 10.1371/journal.pone.0221440 (PMC6701775; doi:10.1371/journal.pone.0221440)

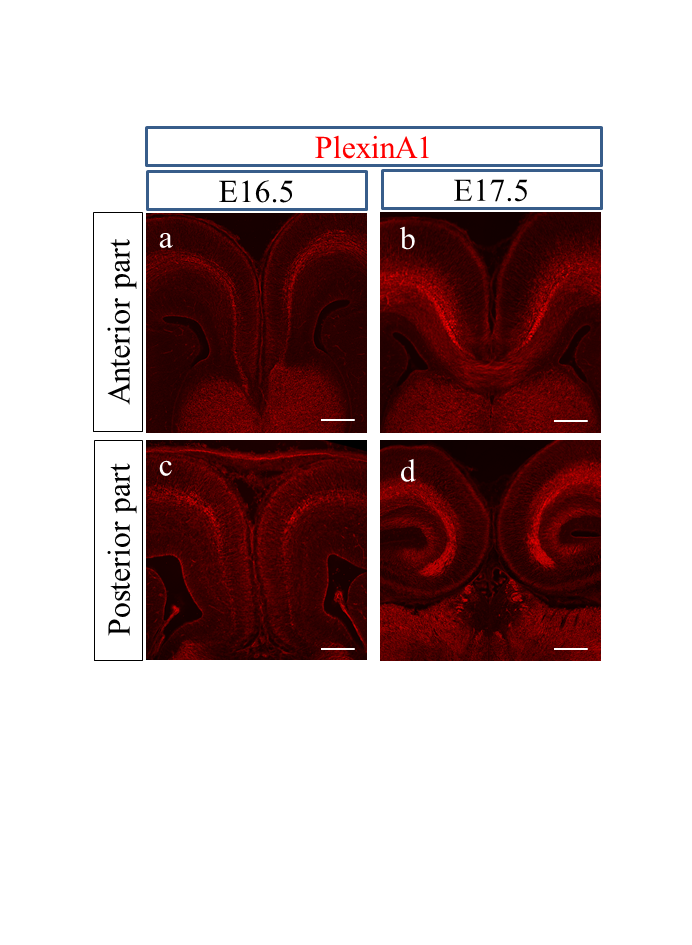

Supplement: S1 Fig — PlexinA1 is expressed in the anterior and posterior part of the WT brain at E17.5. scale bar: 200μm. (TIF) [file pone.0221440.s001.tif]

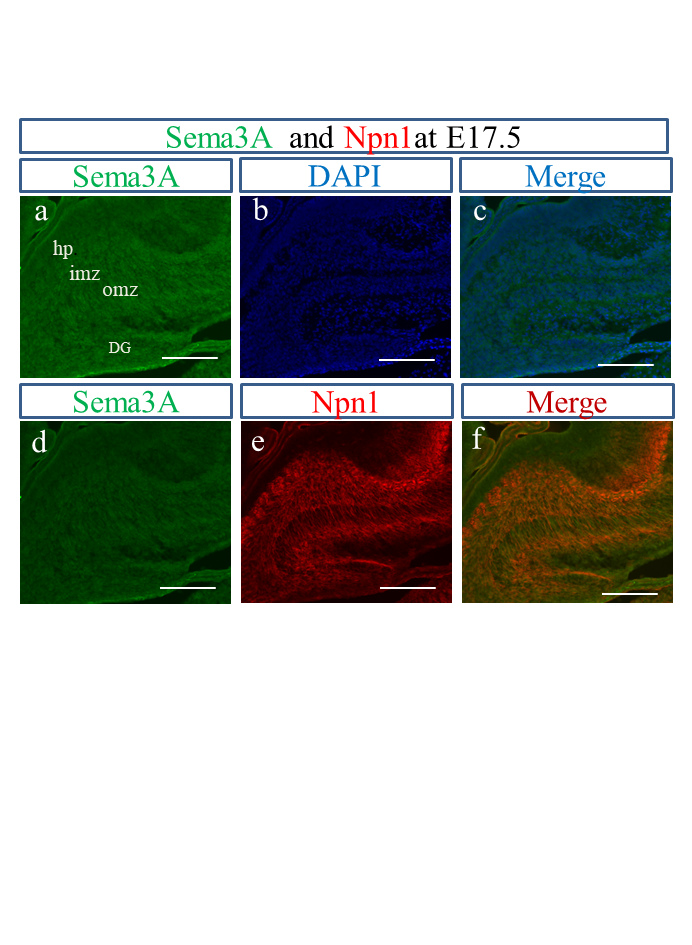

Supplement: S2 Fig — The antibody against Sema3A showed the positive signal in the pial side of the hippocampal plate and inner marginal zone at E17.5. hp: hippocampal plate, imz: inner marginal zone, omz: outer marginal zone, DG: dentate gyrus, scale bar: 200μm. (TIF) [file pone.0221440.s002.tif]

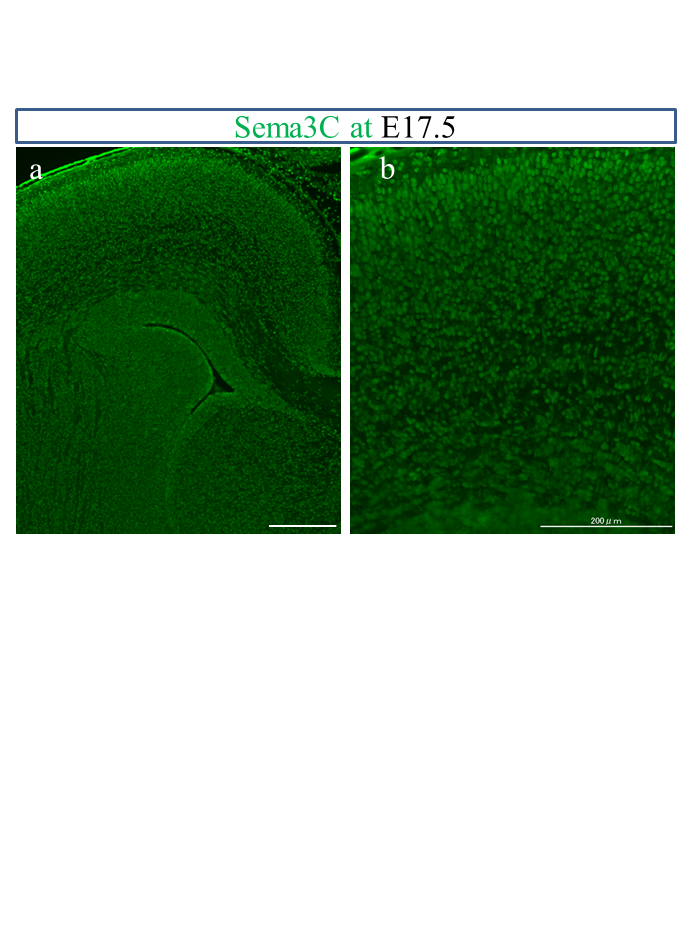

Supplement: S3 Fig — Immunohistochemistry performed with the use of the antibody against Sema3C showed a positive signal in the cortical plate, intermediate zone, and subventricular zone in the developing neocortex on E17.5 (b: higher magnification of a). sacle bar: 200μm. (TIF) [file pone.0221440.s003.tif]

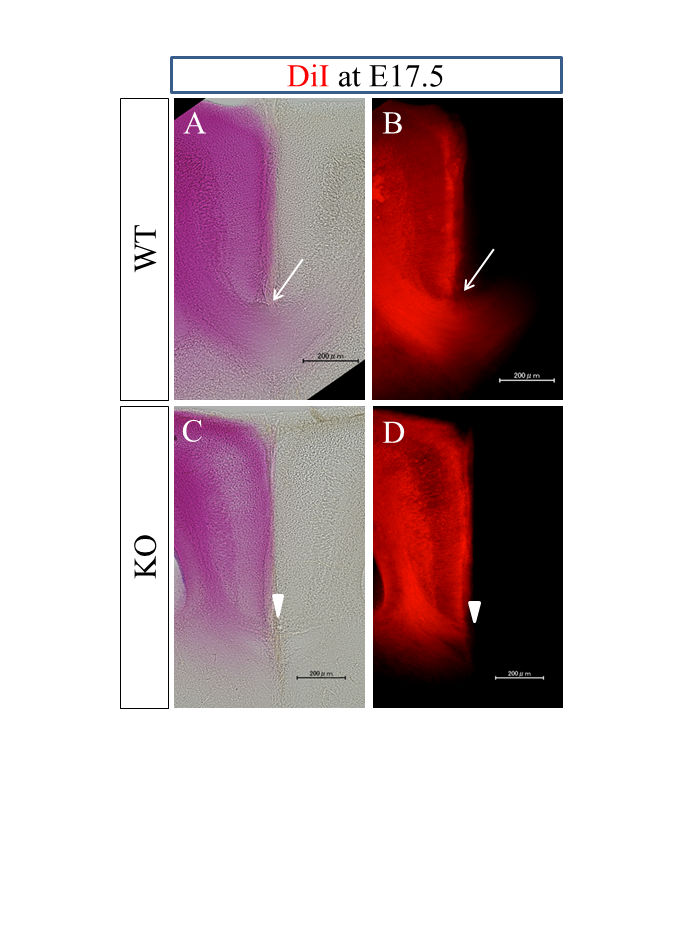

Supplement: S4 Fig — DiI was injected into the cingulate cortex of the right hemisphere of brain sections of WT and PlexinA1 KO mice at E17.5. The images were captured under optical (A and C) and fluorescent (B and D) microscopy. The bundles of callosal axons cross the midline in the contralateral hemisphere of the cerebral cortex in nine out of 10 WT mice (arrows in A and B). In contrast, the callosal axons do not cross the midline in 14 out of 16 PlexinA1 KO mice (arrow heads in C and D). Scale bars: 200 μm. (TIF) [file pone.0221440.s004.tif]

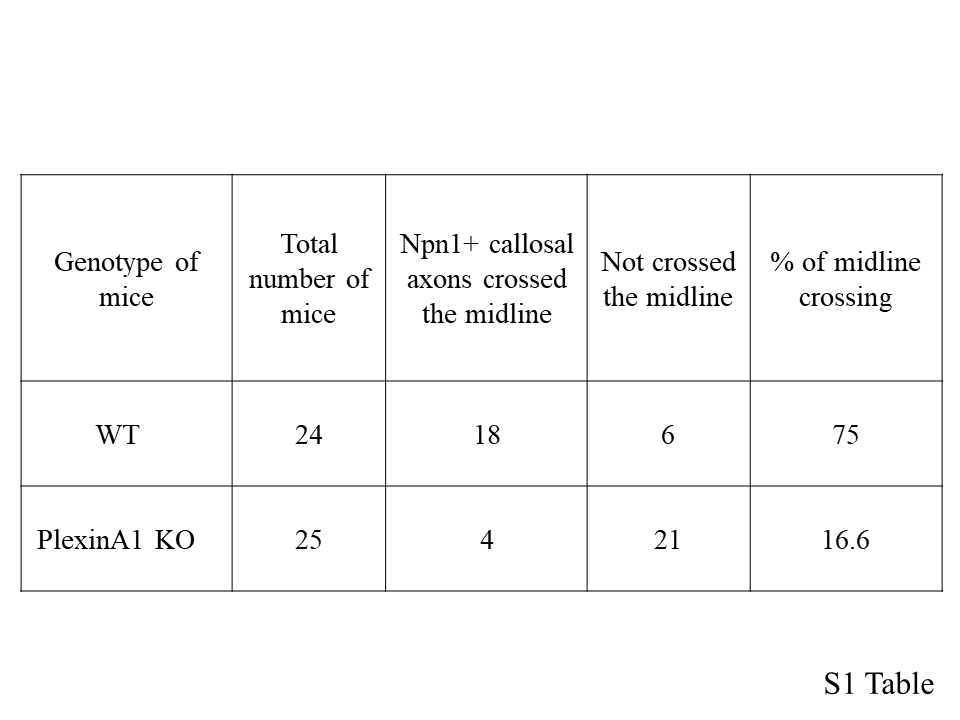

Supplement: S1 Table — In WT mice, the midline crossing of Npn1+ callosal axons is observed in 18 out of 24 mice (75%), and is not detected in six out of 24 mice. In PlexinA1 KO mice, the midline crossing of Npn1+ callosal axons is observed in four out of 25 mice (16.6%), and is not detected in 21 out of 24 mice. The incidence of the midline crossing is significantly lower in PlexinA1 KO mice as compared with that in WT mice (χ2 test, P < 0.05). (TIF) [file pone.0221440.s005.tif]

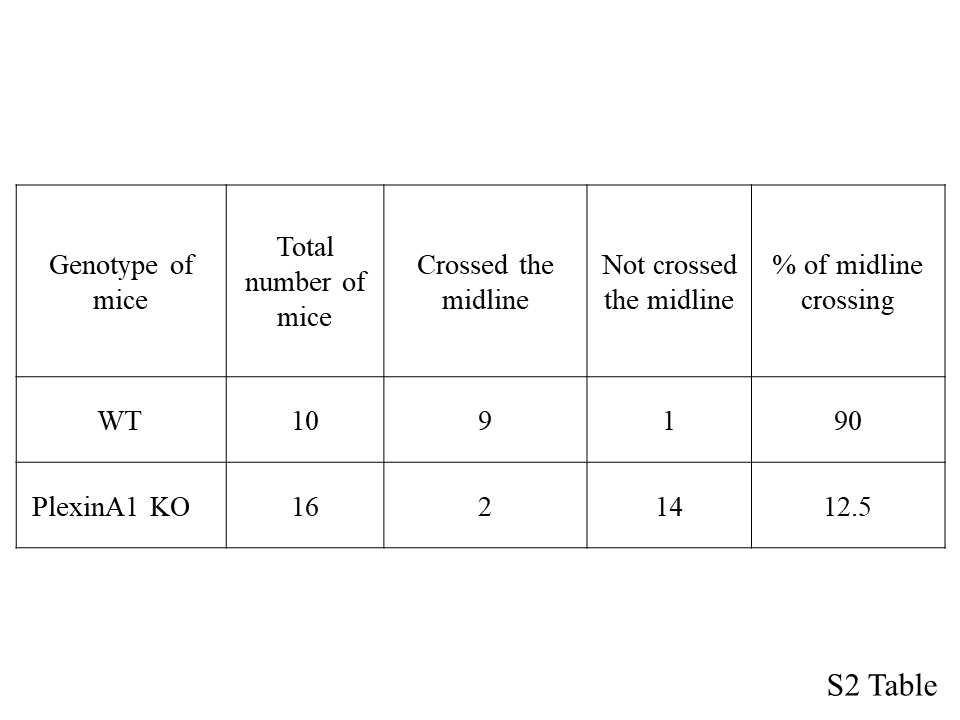

Supplement: S2 Table — In WT, DiI-labeled callosal axons cross the midline in nine out of 10 mice (90%). In PlexinA1 KO mice, DiI-labeled callosal axons cross the midline in two out of 16 mice (12.5%). The midline crossing incidence is significantly lower in PlexinA1 KO mice as compared with that in WT mice (χ2 test, P < 0.05). (TIF) [file pone.0221440.s006.tif]

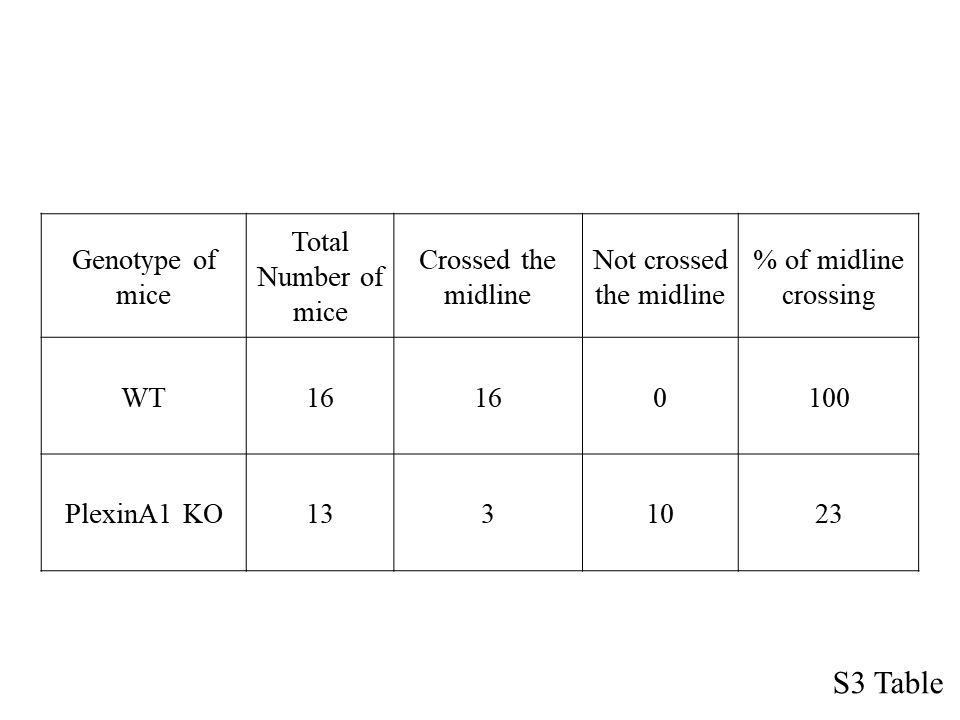

Supplement: S3 Table — In WT, L1CAM+ callosal axons cross the midline in 16 out of 16 mice (100%). In PlexinA1 KO mice, L1CAM+ callosal axons cross the midline in three out of 13 mice (23%). The midline crossing incidence is significantly lower in PlexinA1 KO mice as compared with midline crossing incidence in WT (χ2 test, P < 0.05). (TIF) [file pone.0221440.s007.tif]

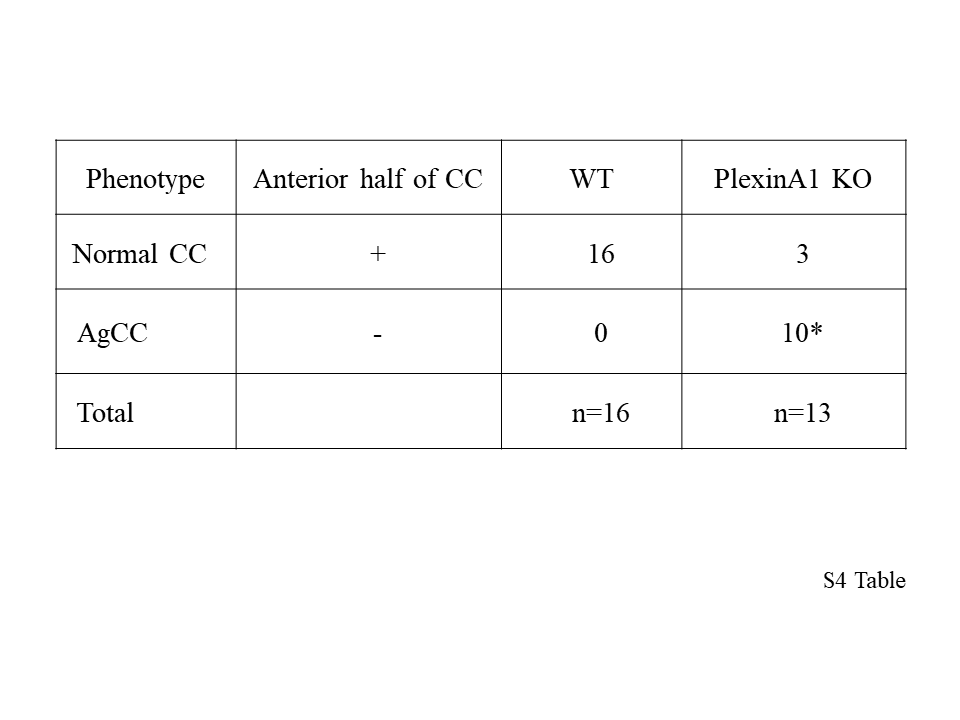

Supplement: S4 Table — Sixteen out of 16 WT mice have normal corpus callosum (CC). In 10 out of 13 PlexinA1 KO mice, agenesis of corpus callosum (AgCC) was detected in the anterior half of the CC. +: callosal axons cross the midline. -: callosal axons do not cross the midline. CC: corpus callosum. AgCC: agenesis of corpus callosum. *χ2 test, P < 0.05. (TIF) [file pone.0221440.s008.tif]
